# What is winter? Modeling spatial variation in bat host traits and hibernation and their implications for overwintering energetics

**DOI:** 10.1002/ece3.7641

**Published:** 2021-08-18

**Authors:** C. Reed Hranac, Catherine G. Haase, Nathan W. Fuller, Meredith L. McClure, Jonathan C. Marshall, Cori L. Lausen, Liam P. McGuire, Sarah H. Olson, David T. S. Hayman

**Affiliations:** ^1^ Molecular Epidemiology and Public Health Laboratory Hopkirk Research Institute Massey University Palmerston North New Zealand; ^2^ Department of Microbiology and Immunology Montana State University Bozeman MT USA; ^3^ Department of Biological Sciences Texas Tech University Lubbock TX USA; ^4^ Conservation Science Partners Truckee CA USA; ^5^ Institute of Fundamental Sciences Massey University Palmerston North New Zealand; ^6^ Wildlife Conservation Society Canada Toronto ON Canada; ^7^ Wildlife Conservation Society Health Program Bronx NY USA; ^8^ Present address: Department of Biology Austin Peay State University Clarksville TN USA; ^9^ Present address: Texas Parks and Wildlife Department Nongame and Rare Species Program Austin TX USA; ^10^ Present address: Department of Biology University of Waterloo Waterloo ON Canada

**Keywords:** hibernation energetics, *Myotis lucifugus*, *Pseudogymnoascus destructans*, white‐nose syndrome, winter duration

## Abstract

White‐nose syndrome (WNS) has decimated hibernating bat populations across eastern and central North America for over a decade. Disease severity is driven by the interaction between bat characteristics, the cold‐loving fungal agent, and the hibernation environment. While we further improve hibernation energetics models, we have yet to examine how spatial heterogeneity in host traits is linked to survival in this disease system. Here, we develop predictive spatial models of body mass for the little brown myotis (*Myotis lucifugus*) and reassess previous definitions of the duration of hibernation of this species. Using data from published literature, public databases, local experts, and our own fieldwork, we fit a series of generalized linear models with hypothesized abiotic drivers to create distribution‐wide predictions of prehibernation body fat and hibernation duration. Our results provide improved estimations of hibernation duration and identify a scaling relationship between body mass and body fat; this relationship allows for the first continuous estimates of prehibernation body mass and fat across the species' distribution. We used these results to inform a hibernation energetic model to create spatially varying fat use estimates for *M. lucifugus*. These results predict WNS mortality of *M. lucifugus* populations in western North America may be comparable to the substantial die‐off observed in eastern and central populations.

## Graphical abstract

White‐nose syndrome disease severity is driven by the interaction between bat characteristics, the cold‐loving fungal agent, and the hibernation environment. Here, we develop predictive spatial models of body mass for the little brown myotis (*Myotis lucifugus*) and reassess previous definitions of the duration of hibernation of this species. Our results provide improved estimations of hibernation duration and identify a scaling relationship between body mass and body fat; this relationship allows for the first continuous estimates of prehibernation body mass and fat across the species' distribution.

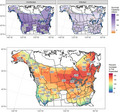

## 1 INTRODUCTION

In temperate zones of North America, hibernating animals, including *Myotis lucifugus* (little brown myotis), bridge resource‐poor winters through energetic budgeting and behavioral changes (Hock, 1951; Ruf & Geiser, 2015; Speakman & Rowland, 1999; Wang, 1978). Survival over winter hibernation depends upon three main facets: (a) the amount of energy stored, primarily in body fat, (b) energetic expenditure (rate of metabolic consumption), and (c) the duration of hibernation (Humphries et al., 2002). Hibernation is composed of bouts of torpor, during which body temperature drops to near ambient temperature to limit heat loss and results in reduced metabolism to restrict the consumption of finite metabolic resources. Torpor is periodically interrupted by energy‐intensive periods of arousal during which hibernators return to euthermic body temperature (Hayman et al., 2010). Hibernators arouse for a variety of proposed reasons (for a review see Carey[, 2019] and citations within), including the need to eliminate metabolic waste, regain water balance, or mate. While arousals represent a small fraction of the total time spent in hibernation, they account for the majority of energy consumed, with a single arousal costing as much as 5% of total overwinter energetic costs (Thomas et al., 1990).

Microclimate selection is critical for hibernators (Boyles et al., 2007). By allowing body temperature to drop during torpor, *M. lucifugus* consumes roughly 80‐fold less energy per unit time due to the relationship between metabolic rate and temperature (Hock, 1951; Speakman & Thomas, 2003). To maximize the utility of these metabolic reductions, bats seek out caves, mines, scree slopes, or other locations generally referred to as “hibernacula” where they can overwinter (Speakman & Thomas, 2003). Relative to the surface landscape, subterranean locations can provide suitable low temperature (i.e., 0–10°C) habitats for hibernators (Thomas & Cloutier, 1992). Microclimate selection will vary greatly based on species‐specific preferences (Haase et al., 2021), and roost selection within the larger hibernaculum critically affects both the frequency of arousals and efficiency of torpor (Czenze et al., 2013; Haase et al., 2019; Humphries et al., 2002). Species such as *M. lucifugus* appear to choose roost locations with stable, humid environments and low temperatures to ameliorate their relatively high rates of evaporative water loss, while other bat species are capable of using more arid, and less thermally stable, roosts as hibernacula (Klüg‐Baerwald et al., 2017; Klüg‐Baerwald & Brigham, 2017). Even so, within a cave or mine system, roost conditions may not remain stationary throughout the duration of hibernation and some bats will relocate within the hibernaculum to seek specific microclimate conditions as their body condition changes (Hayman et al., 2010).

The duration of winter hibernation is another critical determinant to the survival of hibernators. The stimuli that drive immergence (entrance) to and emergence (exit) from hibernation, and their geographic variation, are under‐described (Czenze et al., 2013; Lane et al., 2012; Norquay & Willis, 2014). The duration of winter hibernation presents a strong selective pressure, as longer winters result in shorter growing seasons and less time available for prehibernation fattening (Kunz et al., 1998). Although animals that hibernate at more southern latitudes may be able to capitalize on breaks in the winter weather to opportunistically feed (Thomas et al., 1990), this is not always an option for other latitudes or elevations. Entrances to hibernacula may be blocked by snow, preventing foraging even if breaks in the weather did allow for the re‐emergence of prey species (CLL, unpublished data). Because North American hibernating bats feed on insects, researchers have estimated effective hibernation duration based upon the number of freezing days, with the assumption that freezing temperatures prevent insect availability until spring (Hayman et al., 2016; Humphries et al., 2002). However, some populations of bats may emerge from hibernation while freezing temperatures are still present, suggesting that there may be more complex circadian determinants of emergence times (Johnson et al., 2017). Additionally, site‐level differences, such as slope aspect, foliage cover, and proximity to water, may influence the density of prey insects and their ability to persist on the landscape.

Beyond the normal challenges of hibernation, the epizootic white‐nose syndrome (WNS), caused by the psychrophilic fungus *Pseudogymnoascus destructans*, has increased energetic demands for hibernating bats (Verant et al., 2014; McGuire et al., 2017). The fungal pathogen responsible for the disease has spread rapidly through North America since 2006 and has killed millions of hibernating bats (Frick et al., 2015). Bats can be exposed to the fungus during the swarming period or over hibernation in the hibernacula. Once infected, the fungus grows during hibernation while bat skin temperatures are reduced and immune function is suppressed (Langwig et al., 2015; Langwig et al., 2016; Verant et al., 2012). Although there is still discussion regarding the ultimate cause of mortality in WNS‐impacted bats, the increased frequency of arousals in infected bats, ultimately resulting in the depletion of fat stores prior to the end of the hibernation period and subsequent starvation, presents a likely mechanism (Lilley et al., 2016; Warnecke et al., 2012).

There has been much research on energy consumption over hibernation in multiple bat species (Cryan & Wolf, 2003; Haase et al., 2019; Jonasson & Willis, 2012; McGuire et al., 2014; Thomas et al., 1990; Willis et al., 2006) and across the distribution of single bat species (Hayman et al., 2016; Humphries et al., 2002), but we have yet to determine how the spatial variation in the other two critical parameters governs overwinter survival for hibernators: duration of winter hibernation and amount of fat stores taken into hibernation. Previous models (Hayman et al., 2016) allowed for spatial variation in winter duration; however, the definition of winter duration was made a priori and based solely upon the number of nights with an average temperature below 0°C (Humphries et al., 2002). Similarly, the amount of body fat has generally been fixed as 25%–30% of total body mass in most studies (Haase et al., 2021; Hayman et al., 2016; Humphries et al., 2002). Fat resources are a major determinant of survival (Haase et al., 2019), and thus, this assumption of proportion of body fat warrants review. Here, we used generalized linear and linear models to (a) estimate hibernation duration, (b) relate body mass to prehibernation fat stores, and (c) predict prehibernation body mass and fat across the distribution of *M. lucifugus*. We focused on *M. lucifugus* due to the high impact of WNS on *M. lucifugus* populations, its widespread distribution, and the availability of published data. We predicted that spatial variation in overwinter duration would drive variation of body mass and fat to account for different energy requirements leading to spatial variation in WNS disease outcomes. Finally, we used a mechanistic model of hibernation energetics (Haase et al., 2019) to estimate the total metabolic costs of hibernation with and without the impacts of WNS across the species' distribution of *M. lucifugus*.

## 2 METHODS

### 2.1 Hibernation duration data

We compiled hibernation duration data from literature, publicly available datasets, and our own field data. We also solicited data‐based estimates of hibernation duration from local bat researchers across North America where few records were available from other sources (Table S1, Figure S1). We restricted the study region for this analysis to temperate North America (above the Tropic of Cancer and below the Arctic Circle), although the published range of *M. lucifugus* extends into the Arctic (Fenton & Barclay, 1980). Spatially explicit records of immergence and emergence collected from the literature generally reported the average day of entrance or emergence, although some data were presented only as the duration of hibernation. Where sex‐specific dates were given for a location, dates were averaged as insufficient data existed to complete a sex‐specific analysis.

Acoustic bat recorders (Songmeter SM2+BAT; Wildlife Acoustics) were deployed by Wildlife Conservation Society Canada (WCS‐C) to record bat activity across western Canada between 2008 and 2016. Microphones (either SMX‐US or SMX‐U1) were placed on 12‐ to 18‐foot‐tall telescoping poles above likely hibernation or commuting areas (e.g., riverbanks, cliff ridge tops) starting as early as mid‐August. Acoustic activity was typically recorded throughout the duration of the winter and data were retrieved between mid‐March and mid‐May. Recordings were manually analyzed using AnalookW software (Titley Scientific, Inc.), and customized noise filters were used to pull files containing bat pulses. The occurrence of at least two bat echolocation pulses in a file was required to identify the recording as a “bat pass,” and the number of passes was summed nightly. The start of the hibernation period was defined by the last 3‐night window between August 15 and December 31, in which ≥10 passes were identified, while the end of the hibernation period was defined by the first 3‐night window between March 1 and May 15 in which ≥10 passes were recorded (Lausen & Barclay, 2006). In locations where bat activity was consistently low throughout the year (spring and fall nightly activity often failing to exceed 10 passes), hibernation start and end points were defined by date of the last and first bat recordings, respectively. For sites with multiple years of data, we took the mean immergence and emergence dates.

### 2.2 Prehibernation fat and body mass data

The data required to directly assess the spatial variation in body fat for *M. lucifugus* prior to hibernation do not exist, so we relied upon scaling fat mass with spatially varying body mass. We used body composition datasets obtained by quantitative magnetic resonance (QMR; McGuire & Guglielmo, 2010) measurements that provided both prehibernation fat mass and body mass information from multiple *M. lucifugus* populations, including New York, Vermont (McGuire et al., 2018) and Montana (Haase et al., 2019). We tested for differences in fat among locations using a linear model with fat as a response variable. Given that there was no significant difference among locations, we then fit a linear model with fat as the response and body mass as the predictor variable to predict fat mass from body mass (French, 1985). This fitted relationship then allowed us to predict fat mass at locations where we only had body mass measurements. We gathered additional body mass data from the literature and VertNet (vertnet.org), in which records were filtered to include only those with geographic location, body mass, and recorded between September and December (Table S2, Figure S1).

### 2.3 Prediction of spatially varying hibernation duration and fat mass

Rather than photoperiod‐based metrics, we used the availability of food resources to predict the onset of hibernation, including climatic variables due to the correlation between ambient temperature and insect activity (Mellanby, 1939). The first (herein *Original* model) definition of hibernation duration (Humphries et al., 2002) was the number of days per year where mean nightly temperature from 12 a.m. to 6 a.m. was below freezing. The alternative spatial covariate layers we assumed to be related to the duration of winter were: degrees latitude North (*Northing*), elevation (*DEM*), number of days of frost annually (*Days_frost_*), number of days with an average temperature below freezing annually (*Days_freezing_*), and number of days outside of the growing season annually (*Days_grow_*). Climate data were generated with the ClimateNA v5.10 software package based on methodology described by Wang et al. (2016). All spatial data were re‐projected to the same 1 km^2^ resolution, cropped, and masked to the study extent (Frick & Hijmans, 2017).

All covariates were regressed against the estimated duration of hibernation in univariate models and in multivariate models adjusting for latitude (*Northing*) and/or elevation (*DEM*). Models were assessed through a two‐step process: initial linear models were cross‐validated using Holm's method (Holm, 1979) to identify outliers and generalized linear models were then fit to the modified data set. From initial model fits, externally studentized residuals were converted to *p*‐values and adjusted for multiple testing using Holm's method. Observations with adjusted *p*‐values (<0.5) across multiple models were excluded, and subsequent models refitted using reduced data. Generalized linear models were then fit with the modified dataset, and model selection was assessed by Akaike information criterion (AIC; (Burnham & Anderson, 2002)).

To predict hibernation duration over the study region using the best model selected by AIC, we first assessed model residuals for spatial autocorrelation using Moran's I from the *spdep* R package (Bivand & Wong, 2018). When no autocorrelation was identified, the top model by AIC was predicted across the study extent using the spatial layers to create a continuous estimate of hibernation duration across the species' distribution. If autocorrelation was detected, a spatially weighted generalized linear model was generated using the *glmmfields* package (Anderson & Ward, 2019) for the top model by AIC and then hibernation duration was projected across the species' distribution.

Since these proposed spatial covariates defined the annual inactive period, we also assume that they were likely to drive spatial variation in body mass as bats would need increased metabolic resources to survive longer durations of hibernation. We repeated the above model fitting and selection methods of the same independent variables against the response variable of prehibernation body mass across all data collection locations. We then assessed the top model for spatial autocorrelation and predicted body mass across the species' distribution. Finally, we predicted spatially varying prehibernation fat mass across the species' distribution using the scaling equation fit above.

### 2.4 Prediction of spatially varying hibernation survival

We used a mechanistic hibernation energetics model to estimate the total cost of hibernation (in grams of fat) for a hibernating *M. lucifugus* across its distribution (see Haase et al. [2019] for complete model documentation). The model is dynamic and species‐specific, using metabolic and morphometric parameters to estimate the amount of fat used during hibernation. We used published parameter values for *M. lucifugus* (Haase et al., 2019) with the exception of arousal duration, which was set to 2.2 hr (CLL, unpublished data).

These energetic functions are dependent on temperature and relative humidity of the roost to provide estimates of energy expenditure across a wide range of potential conditions. The temperature and humidity‐dependent growth of *P. destructans* can be included in the model to estimate the metabolic impact of infection on energy consumption as the fungal load increases. We modeled the total energetic costs of hibernation with and without the impacts of *P. destructans* across two hibernaculum microclimate scenarios. First, we used fixed microclimate conditions that assumed a bat could access the preferred optimal hibernaculum microclimate conditions across the species' distribution. We used conditions of 4°C and 98% relative humidity based on observations reported in the literature (Table S3). These microclimatic conditions are thought to provide for the longest possible hibernation duration, a hypothesis supported by recent findings (Haase et al., 2019). Second, we used a spatially explicit model of subterranean temperature conditions to predict the best available (i.e., closest to optimal) temperature at a given location. This approach assumes that bats will select roosts within hibernacula that offer their preferred temperature when possible but will likely tolerate warmer or cooler temperatures when necessary, especially at the range margins. Unfortunately, not much information exists for relative humidity; therefore, we used the optimal 98% relative humidity for both the optimal and best available temperature scenarios.

To estimate the closest available temperature to the optimal temperature at any given location, we used a spatially explicit model of subterranean winter temperatures (McClure et al., 2020). This model estimates subterranean winter temperature should a potential hibernaculum exist based on mean annual surface temperature, site type (cave or mine), distance from the site entrance, and several less influential predictors representing topography, land cover, and presence of water. The model predicts an increase in subterranean temperature with increasing mean annual surface temperature and distance from the site entrance and higher temperatures in mines than in caves. For each 1 km^2^ cell, we estimated minimum and maximum roosting temperatures likely to be present within a hibernaculum using methods described in McClure et al. (2020). We then assigned each raster cell the best available temperature; either the preferred temperature if that temperature was predicted to be available given the minimum and maximum temperature estimations, or the closest temperature available to the preferred roost temperature.

Finally, we estimated the amount of fat required to survive hibernation for each of our 1 km^2^ cells for both healthy and WNS‐impacted bats by using the dynamic hibernation energetics model and our spatially varying predictions of hibernation duration and fat. We determined whether an average bat could survive hibernation for each cell by subtracting the predicted fat required to survive the hibernation duration from the fat available prior to hibernation. Positive values indicate a bat's ability to survive hibernation with excess fat, while negative values indicate the depletion of fat stores prior to the end of the hibernation period. We compared the predicted amount of fat required by healthy bats against the fat required by infected bats to estimate the relative increase in energetic costs of *P. destructans* on *M. lucifugus* as a percentage (the difference between resources required to survive hibernation when infected and when healthy in grams of fat, divided by grams of fat required to survive hibernation as healthy bat, multiplied by 100). We also translated the amount of fat “leftover” after the conclusion of hibernation into days posthibernation, that is, how long the bat could continue hibernating, for both healthy and infected bats.

All analyses were performed in R (R Development Core Team, 2009), with spatial handling tools from *raster* (Hijmans, 2016) and *sp* packages (Pebesma & Bivand, 2005).

## 3 RESULTS

### 3.1 Hibernation duration

**FIGURE 1:**
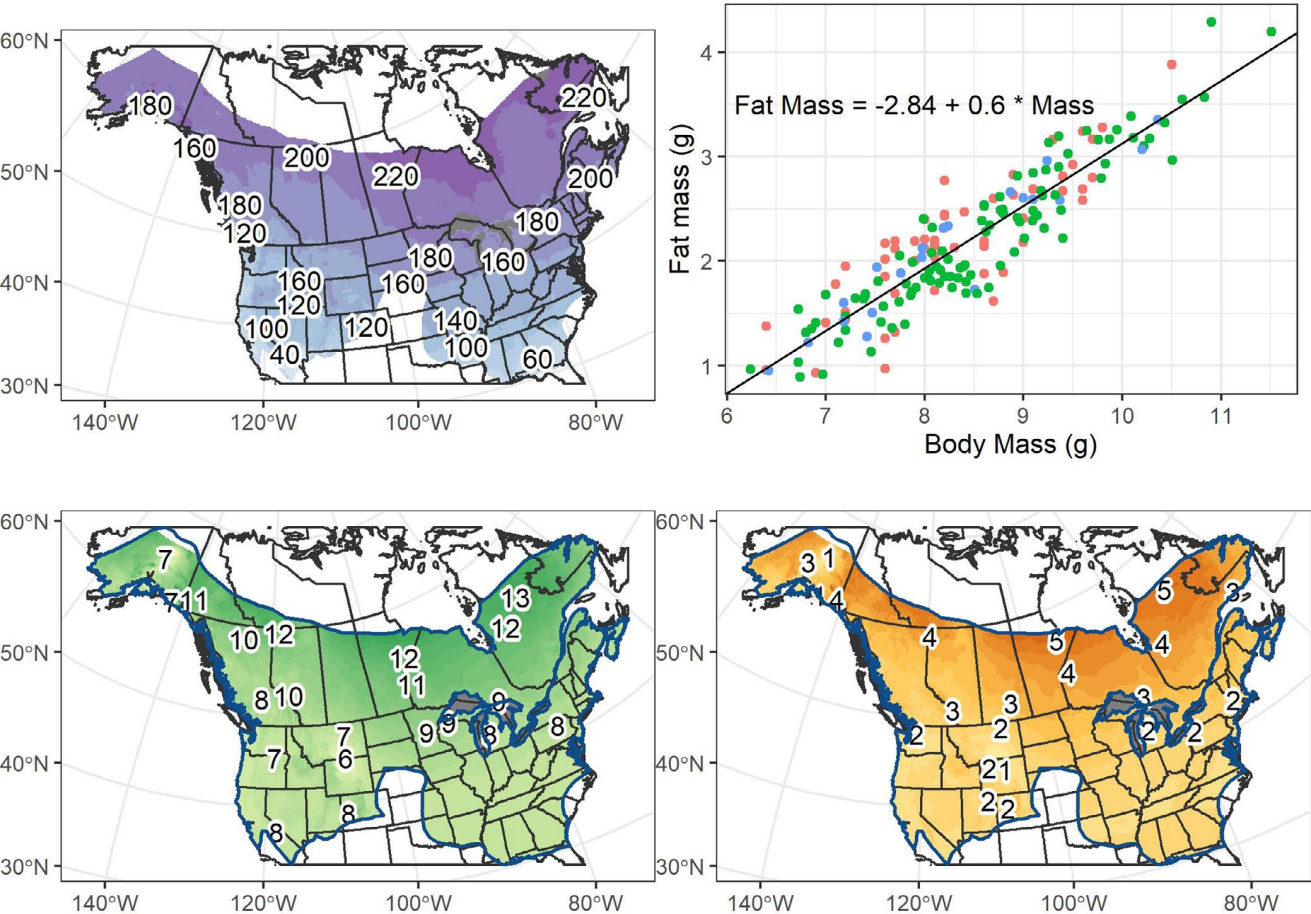
Predicted hibernation duration, fat, and body mass for *Myotis lucifugus*. Top left: Predicted hibernation duration (days) across temperate North America for *M. lucifugus*. Top right: Linear relationship between body fat and body mass. Each point represents an individual bat and colors indicate the state of each record (Montana = red, New York = green, Vermont = blue). Bottom left: Predicted prehibernation body mass of *M. lucifugus* (g) across the species' distribution. Bottom right: Predicted prehibernation fat resources (g) from the linear relationship between fat mass and body mass. Numbers define changes in contours

Latitude (*Northing*; *β* = −0.22, *p*‐value = 0.85), elevation (*DEM*; *β* = −0.03, *p*‐value = 0.04), and the number of days in frost (*Days_frost_*; *β* = 0.83, *p*‐value < 0.001) best predicted hibernation duration across the study area (*AICc* = 489.29; Table S4). There was no spatial correlation of these predictions (*Moran's I* = −0.0036, *p*‐value = 0.354), and thus, no spatial corrections were needed. The selected model outperformed the original a priori estimate of the duration of winter (*Original*, ∆AIC 19.06) (Figure 1).

**FIGURE 2:**
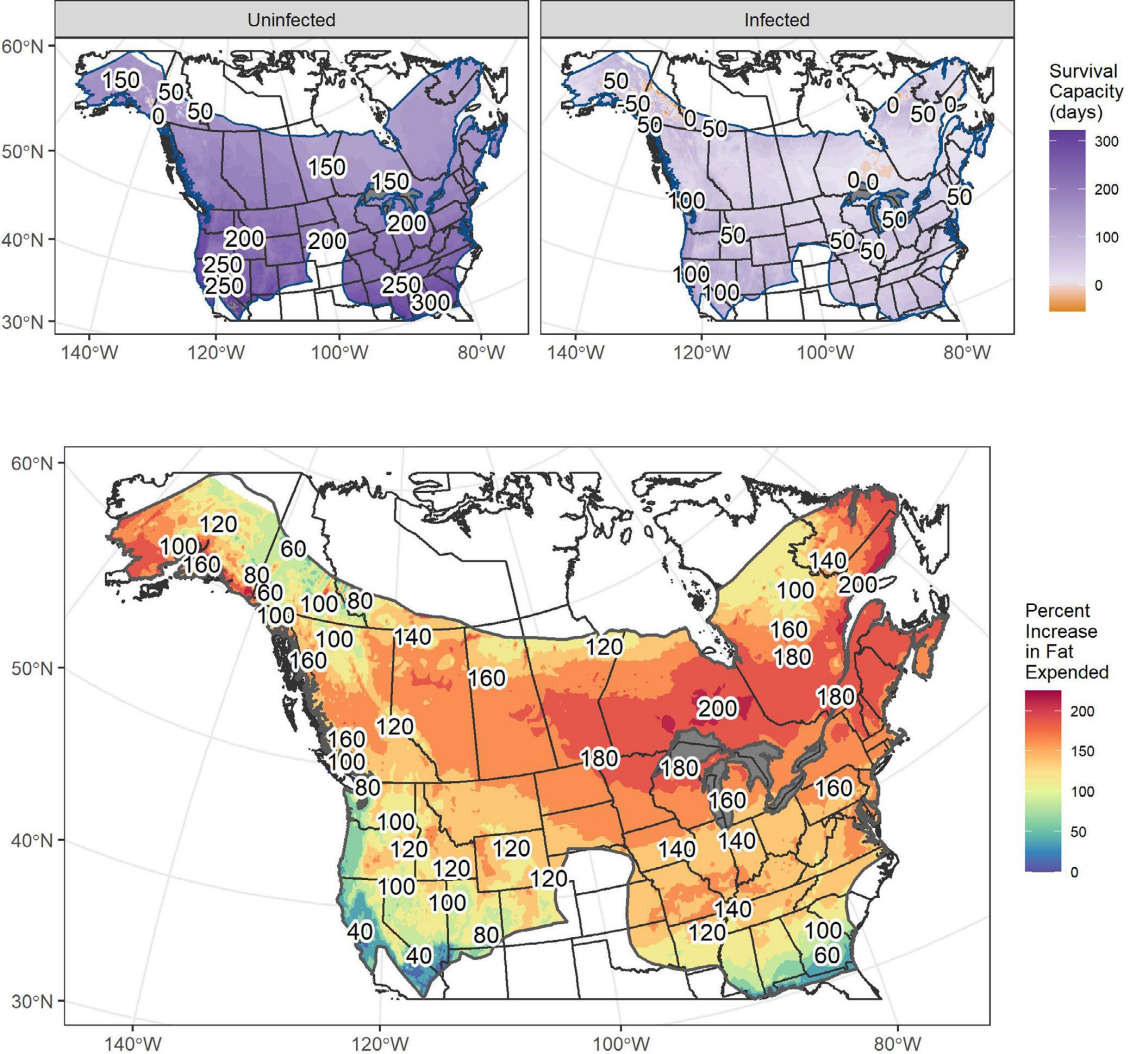
Top row: Predicted survival (days) for healthy (top left) and infected (top right) *Myotis lucifugus* hibernating at the best available temperatures (°C) in caves or mines and 98% relative humidity. Survival was calculated by subtracting the maximum days in hibernation that an individual can tolerate based upon prehibernation fat stores from the predicted duration of hibernation. Positive values (purple) suggest bats will likely survive the duration of hibernation under those conditions while values below 0 imply that the fat stores were insufficient for survival. Values depicted on the maps indicate the predicted maximal number of days that a bat could continue to hibernate beyond the predicted duration of winter. Bottom: Relative increase (%) in fat stores used over predicted hibernation duration when infected with *Pseudogymnoascus destructans* compared with healthy bats when hibernating at the best available temperature predicted to occur within caves and mines at 98% relative humidity

Given the best model, median‐predicted hibernation duration was ~179 days (*mean* = 169.16, *sd* = 45.36). Maximum hibernation duration across the study extent was estimated at ~289 days in the upper portions of Manitoba, Ontario, and Quebec, 45 days longer than the longest observation in the training data (from Manitoba, Canada, 19; Figure 2). Despite this, 95% of all cells had a predicted hibernation duration below 225 days, and only 5% were below 80 days (Table S5).

### 3.2 Body mass

Prehibernation fat stores were strongly related to body mass (*β* = 0.597, *F_1,171_* = 826.7, *p* = <0.001, adjusted *R* ^2^ = 0.8276; Figure 2). Latitude (*Northing*; *β* = −0.08, *p*‐value = 0.07) and the number of days below freezing (*Days_freeze_*; *β* = 0.04, *p*‐value < 0.001) best predicted fat mass across the species' distribution (*AICc* = 162.57; Table S4). However, the model including latitude, number of annual days below freezing, and elevation performed similarly (*ΔAICc* < 2; Table S4).

Given the best model, the median predicted prehibernation body mass across the species' distribution of *M. lucifugus* was 8.65 g (*mean* = 9.14 g, *sd* = 1.84 g) and 95% of the cells predicted values between 7.04 g and 12.52 g. Median prehibernation fat stores were predicted at 2.32 g (*mean* = 2.61 g, *sd* = 1.10 g) with 95% of cells predicting available fat available ranging between 1.36 and 4.63 g.

### 3.3 Overwinter hibernation survival

Overall, our energetic model predicted nearly ubiquitous survival of uninfected bats throughout the range of *M. lucifugus* with <0.0001% of cells falling below the threshold for survival. Our results found that 95% of all uninfected bats roosting at 4°C and 98% relative humidity during hibernation would only require 0.21–0.60 g of fat to survive the duration of hibernation (*median* = 0.48 g, *mean* = 0.45 g, *sd* = 0.12 g, Figure S2). When considering the amount of fat bats had prehibernation, the median bat emerged with 1.85 g of body fat remaining (*mean* = 2.16 g, *sd* = 1.01 g) and the heaviest 95th percentile of bats had up to 4.05 g of fat remaining. Considering these residual fat values in the terms of days spent in hibernation, the median bat would have sufficient fat resources to survive an additional 181 days (*mean* = 190.83 days, *sd* = 45.35 days) and those bats with <4 g of fat remaining could be capable of surviving another 280 days in those optimal roosting conditions.

When *P. destructans* infection was included in the optimal roosting conditions, the median value of fat required to survive hibernation was increased by 0.7–1.21 g (*mean* = 1.16 g, *sd* = 0.45) and the residual fat values dropped to a median of 1.22 g (*mean* = 1.45 g, *sd* = 0.76). In total, 95% of bats were predicted to emerge with between 0.61 and 2.93 g of fat remaining after the hibernation. Translating the fat values into days, the median value was reduced ~135 to 45.63 days (*mean* = 55.41 days, *sd* = 45.35 days). Mortality of bats prior to the end of the hibernation period was predicted in 4.82% of the cells where the survival of hibernating bats fell <0, visible in the northeastern provinces of Canada (Figure S2).

Most of the predicted best available subterranean hibernacula were expected to have available temperatures at or above 4°C. Despite this, 32.64% of cells fell below that temperature and 6.44% of cells fell below the lower critical temperature of 2°C that *M. lucifugus* defends during hibernation (Figure S3). Despite these lower than optimal temperatures, *M. lucifugus* was predicted to survive across most of its distribution, with only 0.5% of cells predicted to fall below the survival threshold—primarily around Denali National Park in Alaska. Median fat required to survive hibernation as an uninfected bat dropped 0.71–1.11 g (*mean* = 1.41 g, *sd* = 0.93) with a nearly identical 95% interior range. When including infection with *P. destructans,* the hibernation energetics model predicted the median fat required for hibernation increased 0.72–1.21 g, (*mean* = 1.20 g, *sd* = 0.40 g). Like optimal roosting conditions, infection resulted in 4.74% of cells falling below the survival threshold.

Although neither of the considered hibernation conditions predicts large areas to result in mortality, the increased percent of fat needed to hibernate with *P. destructans* highlights the metabolic consequences of infection (Figure S4). At 4°C and 98% relative humidity, bats were predicted to expend a median of 154% more body fat resources to hibernate while infected for the same winter duration (Table S4). Overall, 95% of infected bats are predicted to increase their metabolic expenditure between ~66% and 195% compared with their healthy counterparts. Hibernating at the predicted best available temperature suggested similar increases in the energy expended; however, the geographic distribution of where the greatest increases occurred were different than those observed under static preferred conditions.

## 4 DISCUSSION

Here, we examined the spatial variation in hibernation duration and estimated prehibernation fat stores across North America and applied these estimates to an updated model of hibernation energetics (Haase et al., 2019) to estimate the overwinter fat necessary for *M. lucifugus* to survive the hibernation. By comparing the required fat and the fat available, we predicted survival of *M. lucifugus* across its distribution for two different ecological situations: one in which bats roost within their most preferred conditions and one in which they roost in the best conditions predicted to be available. Finally, we modeled the possible impact of *P. destructans* infection to understand the implications for *M. lucifugus* populations where bats have yet to be impacted by WNS.

Winter hibernation duration is a key determinant of the overwintering survival for any hibernating species. While local variation in winter onset due to unique landscape features may create refugia where bats may persist later than or emerge from hibernation earlier than average, the lack of any previous broad‐scale estimates for this critical variable highlights the need for this study. Prior work (Hayman et al., 2016; Humphries et al., 2002) had defined the hibernation period a priori as the yearly number of nights with a mean nightly temperature below freezing. Our results suggest that substantial improvements in the estimation of hibernation duration can be made by including elevation, latitude, and the number of days with frost. The number of days of frost, rather than the number of days below freezing, and the counter‐intuitive negative coefficients for both *Northing* (−0.45) and *DEM* (−0.04) in the model suggest that there is more nuance to the relationship between bats and low temperatures than currently understood. Our model is potentially biased in part by the over‐representation of Canadian data, low elevation sites, and the collinearity of elevation and latitude with other explanatory variables. Notably, however, when included in univariate models, the coefficient signs regress as expected with the coefficients increasing with latitude and elevation; yet, the univariate models do not predict hibernation duration as well and had higher AIC scores. Because we were interested in prediction, we kept the best model by AIC; however, clearly more work is required to understand what predicts overwinter duration in bats. Also, there were few estimates of hibernation duration from the western‐most US states and the more southern latitudes within the species' distribution. These factors likely impacted model results in unpredictable ways and highlight the need for additional data collection.

Species such as *M. lucifugus* may not hibernate across their broadest summer distribution, but likely seek out locations with more favorable conditions to overwinter. The maximum winter hibernation duration predicted within the distribution of *M. lucifugus* was a month and a half longer than the longest observation within our dataset, and this did not consider the portion of the species' distribution that extended into the Arctic Circle. In all probability, bats likely do not overwinter within these regions for multiple reasons. First, while our survival models predict that an uninfected bat could survive the longest predicted winter hibernation, the necessary roosting microclimate conditions may not exist on the landscape (McClure et al., 2020; Perry, 2013). A series of complex interactions between surface features (e.g., slope, aspect, elevation, suitable crevices) or cave features (e.g., number of entrances, air flow, depth) ultimately define when and where suitable hibernacula conditions exist (McClure et al., 2020; Perry, 2013). These site‐level determinants of microclimate conditions make it difficult to define a relationship between landscape‐level features and the available subterranean conditions and create challenges in attempting to predict where suitable hibernacula conditions exist (McClure et al., 2020). Despite this, our use of modeled subterranean temperatures offers an improvement over assuming either static optimal conditions or mean annual surface temperature used in Hayman et al. 2016. Second, and perhaps more importantly, regions where extended winters reduce the summer active period to <100 days likely create significant challenges to reproductive success. With a gestation period of ~60 days (Kurta et al., 1989; O'Farrrell & Studier, 1973), a female bat would be hard pressed to gestate, nurse, and wean young, while still allowing the young of the year time to fatten sufficiently to survive such an extended hibernation period. These results highlight the fact that only a subset of summer distributions may be suitable for overwinter survival, an idea rarely considered in the definition of bat species' distributions.

Localized clines in body size and body mass of *M. lucifugus* have previously been recorded (Lacki et al., 2015; Lausen et al., 2008). When we compare more detailed metrics of body composition (lean mass and body fat content), however, these clines are better understood: the lean mass of bats generally stays consistent, while the difference in body mass is due to increases in fat. Our model selection suggests that variation in the duration of winter hibernation may in part drive variation in both body mass and fat stores across the range of the species. The relationships between mass (and thus fat) and latitude and days below freezing also suggest stronger selection pressure for heavier bats in more extreme conditions. While still useful, the relationship between the selected abiotic variables and body mass of bats showed strong spatial autocorrelations among residuals, and there may be additional continental‐scale drivers or local determinants not investigated in this study including diversity of prey species and competition between bat species.

The scaling relationship identified between prehibernation fat stores and body mass is a departure from the fixed 30% value used in previous energetic modeling studies (Hayman et al., 2016; Humphries et al., 2002) and is supported by contemporary findings (Cheng et al., 2019). More localized body mass–body fat relationships may exist, yet without increased data resolution drivers of the true relationship will remain difficult to assess. Here, we assumed bats did not forage during hibernation, but some bats (primarily from southern hibernacula) have been known to forage over winter (Bernard & McCracken, 2017). Without data, we were required to assume bats relied exclusively on prehibernation fat stores.

For our modeled roost conditions, virtually all uninfected *M. lucifugus* could survive the estimated duration of winter. Observed rates of survival among uninfected overwintering bats are high (Boyles & Brack, 2009) and bats are likely capable of overwintering across most of their summer distribution where suitable hibernacula exist. These results are an improvement over previous models which did not predict survival where hibernation duration >6 months (Hayman et al., 2016) despite our prediction that some 51% of the study extent may experience winters longer than that. In previous models, roosting microclimatic space was derived exclusively from surface metrics (i.e., mean annual surface temperature and relative humidity). Our use of a static, optimal roosting scenario served as a baseline for a best available temperature scenario and provided a more biologically relevant conditions for *M. lucifugus*, as bats are known to preferentially roost in these conditions when available (Haase et al., 2019; Thomas & Cloutier, 1992). In areas where WNS has devastated hibernating colonies of *M. lucifugus*, available microclimates are often warmer with temperatures reaching 10°C (Perry, 2013). This warmer microclimate has a significant impact on energy expenditure during hibernation (Figure S4). Despite this, our predictions for overwinter survival using estimates of the best roosting temperatures available suggest that survival may still be possible, if bats use the coldest areas within the cave or mine system. In a rare ray of hope, all of the hibernation temperatures that we recorded in the West were well below the 10°C mark, with hibernating *M. lucifugus* in northeastern Alberta and Northwest Territories roosting at about 2°C and 100% relative humidity (CLL, unpublished data). While the high relative humidity is generally beneficial to the growth of *P. destructans* (and thereby a promoter of WNS pathology), the cooler temperatures may slow fungal growth in comparison to the warmer roosts of the Eastern United States.

Our model predicted uninfected bats to emerge from hibernation with remaining fat quantities far greater than the amount of fat thought to be needed to hibernate for the entire duration of winter. Some of this may be an artifact of our modeling, as we may be missing additional energetic costs such as flight during the arousal periods or sex‐related differences, and we did not model deviance from the most optimal arousal patterns that can result from arousals of individuals sharing the hibernacula (neighbor‐initiated arousals; Czenze et al., 2017; Hayman et al., 2010; Jonasson & Willis, 2012; Turner et al., 2015). Alternatively, remaining fat stores may be retained as a buffer against adverse abiotic conditions experienced after emergence, especially among females that undergo pregnancy immediately upon exit from hibernation (Czenze et al., 2017; Frick et al., 2010a; Johnson et al., 2017) and whose reproductive success stands to benefit from a longer growing season. Early emergence would provide a selective advantage to the young of the year, as even one or two extra weeks foraging on the landscape could increase fat stores, making them more prepared for hibernation (Reynolds & Kunz, 2000). Currently, little is known about the energetic demands of the emergent bats as they return to the landscape and accurate parameterization of this factor could significantly change our definition of survival capacity and increase our estimates of WNS‐related mortality.

Modeled hibernation survival predictions of bats infected with *P. destructans* at either 4°C and 98% relative humidity or using the best available temperature do not match observations from the Eastern United States where mass mortality events have occurred due to WNS (Blehert et al., 2009; Frick et al., 2010b; Frick et al., 2015). While our analysis did demonstrate infection with *P. destructans* dramatically increases the amount of energy expended during hibernation, nearly 95% of all cells analyzed predicted survival despite infection. Interestingly, however, the areas with the greatest increase in hibernation energy expenditure using the best available temperature were much more in line with regions where bat populations have experienced the greatest mortality.

Irrespective of the absolute values for survival predicted within this work, our modeling predicts that *M. lucifugus* populations where WNS is currently absent, especially those along the Rocky Mountains, Alberta, British Columbia, and Alaska will require similar increases in energetic expenditure as populations currently impacted by WNS. Thereby, if the increase in energy expenditure results in the same pattern of mortality, *M. lucifugus* populations in western North America may be expected to suffer mortality events similar to those experienced in the eastern and central populations (Frick et al., 2010b; Frick et al., 2015). The long duration winters, especially in northern British Columbia, northern Alberta, Alaska, Yukon, Northwest Territories, and portions of the Rocky Mountains, may result in severe WNS‐associated pathology and disruption to hibernation physiology, although use of cool available hibernacula microclimates in some of these areas may slow fungal growth (Reeder et al., 2012; Verant et al., 2014). Identifying and describing roosting microclimates in these areas will allow for better predictive models at a regional scale. Some northern hibernacula may provide a refugia for *M. lucifugus* infected with WNS; however, this cannot be defined until the availability of microclimates and spring energy requirements are quantified. For example, if gleaning of arthropod prey in spring is typically required for successful reproduction in northern latitude *M. lucifugus* (Kaupas & Barclay, 2017; Talerico, 2008), then the extra energy expenditure associated with gleaning (Norberg et al., 1987) may require additional fat stores and WNS‐related mortality rate may in fact be higher than predicted. Additionally, even if WNS‐related mortality is not directly observed, reproductive success may decline resulting in more long‐term population decline.

The outputs of the energetic model that we applied are sensitive to a number of parameters and assumptions (Haase et al., 2019). The hibernation energetic model is largely derived from first principles and deviations in parameters, especially those defining the frequency or duration of arousal (cluster‐based arousal, partial arousals, disturbances, etc. 8) or increasing these costs (e.g., increased number of mid‐winter flights or disease pathophysiology), have the potential to alter the amount of resources required to survive hibernation. Roost microclimates are important to survival predictions and impact bat energetic and fungal growth dynamics (Marroquin et al., 2017), and further, study is required to understand how relative humidity may vary across the landscape and within hibernacula.

Overall, this work represents an effort to iteratively refine both individual and landscape models of bat hibernation physiology and the impacts of WNS on bat populations. By specifically addressing the spatial heterogeneity of both abiotic phenomena and host traits, we offer new insights into the potential impacts of WNS on *M. lucifugus* as *P. destructans* continues to spread through western North America.

## CONFLICT OF INTEREST

The authors claim no conflict of interest.

## AUTHOR CONTRIBUTIONS

**C. Reed Hranac:** Conceptualization (lead); Formal analysis (lead); Methodology (lead); Validation (lead); Visualization (lead); Writing‐original draft (lead). **Catherine G. Haase:** Data curation (lead); Writing‐review & editing (equal). **Nathan W. Fuller:** Data curation (lead); Writing‐review & editing (equal). **Meredith L. McClure:** Methodology (equal); Visualization (equal); Writing‐review & editing (equal). **Jonathan C. Marshall:** Formal analysis (supporting); Methodology (supporting). **Cori L. Lausen:** Conceptualization (equal); Funding acquisition (equal); Project administration (equal). **Liam P. McGuire:** Conceptualization (equal); Funding acquisition (equal); Project administration (equal); Writing‐review & editing (equal). **Sarah H. Olson:** Conceptualization (equal); Funding acquisition (lead); Project administration (lead); Supervision (lead); Writing‐review & editing (equal). **David T. S. Hayman:** Conceptualization (equal); Funding acquisition (lead); Investigation (equal); Project administration (equal); Supervision (equal); Writing‐review & editing (equal).

## ETHICAL APPROVAL

All procedures were approved by the Texas Tech University Institutional Animal Care and Use Committee (protocol 16031‐05) and permits from the Montana Department of Fish, Wildlife & Parks (permits 2016‐104, 2017‐018, and 2018‐008).

## Supporting information

Supplementary Material

## Data Availability

All data pertaining to the work are available publicly, included as supplementary materials with this publication or available by request. All custom code for this project is available at github.com/cReedHranac/winTor, and the energetic model code is available from github.com/cReedHranac/batwintor.
