# Supplementary material for: What is winter? Modeling spatial variation in bat host traits and hibernation and their implications for overwintering energetics
Source: Ecol Evol. 2021 Aug 18;11(17):11604–14. doi: 10.1002/ece3.7641 (PMC8427580; doi:10.1002/ece3.7641)
Supplement: Supplementary file 1 — Supplementary Material [file ECE3-11-11604-s001.docx]

**Supplemental Information**

**What is winter? Modelling spatial variation in bat host traits and hibernation and their implications for overwintering energetics**

C. Reed Hranac^1¥^, Catherine G. Haase^2+^, Nathan W. Fuller^3$^, Meredith L. McClure^4^, Jonathan C. Marshall^5^, Cori L. Lausen^6^, Liam P. McGuire^3^^, Sarah H. Olson^7^, David T. S. Hayman^1^

^1^ Molecular Epidemiology and Public Health Laboratory, Hopkirk Research Institute, Massey University, Palmerston North, New Zealand

^2^ Department of Microbiology and Immunology, Montana State University, Bozeman, USA.

^3^ Department of Biological Sciences, Texas Tech University, Lubbock, USA

^4^ Conservation Science Partners, Truckee, USA

^5^ Institute of Fundamental Sciences, Massey University, Palmerston North, New Zealand

^6^ Wildlife Conservation Society Canada, Toronto, Canada

^7^ Wildlife Conservation Society, Health Program, Bronx, USA

**Corresponding author:* [crh244@gmail.com](mailto:crh244@gmail.com)

^¥^Current affiliation: Colorado Department of Public Health and Environment, Healthcare Associated Infections and Antimicrobial Resistance Program, Denver, USA

^+^Current affiliation: Department of Biology, Austin Peay State University, Clarksville, USA

^$^Current affiliation: Texas Parks and Wildlife Department, Nongame and Rare Species Program, Austin, USA

^Current affiliation: Department of Biology, University of Waterloo, Waterloo, Ontario, Canada

**Table S1.** **Winter hibernation duration data for *Myotis lucifugus.*** Location data was coerced to the centre of the raster cell to protect hibernaculum locations. If years were not provided, some dates were given arbitrary years to calculate duration.

| Latitude | Longitude | Start | End | Duration | | Reference | | |  |
| --- | --- | --- | --- | --- | --- | --- | --- | --- | --- |
| 53.12 | -99.19 | 15/09/2018 | 15/05/2019 | 242 | | Czenze and Willis 2015 | | |  |
| 51.44 | -97.38 | 13/09/2018 | 15/05/2019 | 244 | | Norquay and Willis 2014 | | |  |
| 53.12 | -99.19 | 15/09/2018 | 15/05/2019 | 242 | | Jonasson and Willis 2012 | | |  |
| 59.75 | -112.20 | 15/10/2018 | 15/05/2019 | 212 | | Reimer et al. 2014 | | |  |
| 44.93 | -110.67 | 15/10/2018 | 10/4/2019 | 177 | Johnson et al. 2016 | | | | |
| 38.83 | -92.29 | NA | NA | 83 | Brack Jr. and Twente 1985 | | | | |
| 36.61 | -83.66 | 4/12/2018 | 1/4/2019 | 118 | Hayman et al. 2017 | | | | |
| 58.30 | -134.40 | 24/10/2018 | 31/03/2019 | 158 | Karen Blejwas | | | | |
| 61.00 | -135.00 | 30/09/2018 | 18/04/2019 | 200 | Tom Jung | | | | |
| 59.60 | -133.60 | 15/10/2018 | 24/03/2019 | 160 | Cori Lausen | | | | |
| 58.90 | -125.80 | 21/10/2018 | 15/04/2019 | 176 | Cori Lausen | | | | |
| 59.40 | -126.10 | 7/10/2018 | 7/4/2019 | 182 | Cori Lausen | | | | |
| 60.00 | -111.88 | 15/10/2018 | 15/04/2019 | 182 | Sharon Irwin and Cori Lausen | | | | |
| 53.80 | -116.50 | 15/09/2018 | 15/05/2019 | 242 | Cori Lausen | | | | |
| 43.24 | -73.51 | 10/10/2018 | 15/05/2019 | 217 | Davis and Hitchcock 1965 | | | | |
| 60.96 | -117.33 | 5/10/2018 | 20/04/2019 | 197 | Joanna Wilson | | | | |
| 47.12 | -111.00 | NA | NA | 180 | Haase et al. 2019 | | | | |
| 51.75 | -124.72 | 15/10/2015 | 21/03/2016 | 158 | WCS-C Recorder | | | | |
| 51.52 | -122.29 | 21/10/2015 | 27/03/2016 | 158 | WCS-C Recorder | | | | |
| 49.12 | -116.63 | 6/11/2012 | 27/04/2013 | 172 | | | WCWCS-C Recorder |  |  |
| 55.20 | -129.10 | 28/10/2015 | 1/3/2016 | 125 | | | WCWCS-C Recorder |  |  |
| 49.01 | -119.50 | 1/12/2012 | 11/3/2013 | 100 | | | WCWCS-C Recorder |  |  |
| 53.88 | -124.59 | 25/09/2015 | 30/03/2016 | 187 | | | WCWCS-C Recorder |  |  |
| 51.50 | -122.30 | 22/10/2015 | 29/03/2016 | 159 | | | WCWCS-C Recorder |  |  |
| Latitude | Longitude | Start | End | Duration | | | Reference |  |  |
| 49.02 | -118.34 | 13/12/2010 | 4/3/2011 | 81 | | | WCS-C Recorder |  |  |
| 55.24 | -127.67 | 11/10/2014 | 8/4/2015 | 179 | | | WCS-C Recorder |  |  |
| 54.30 | -129.40 | 4/10/2014 | 16/03/2015 | 163 | | | WCS-C Recorder |  |  |
| 49.91 | -116.90 | 20/11/2013 | 27/03/2014 | 127 | | | WCS-C Recorder |  |  |
| 49.30 | -116.66 | 28/12/2014 | 4/3/2015 | 66 | | | WCS-C Recorder |  |  |
| 54.41 | -128.52 | 3/11/2015 | 5/3/2016 | 123 | | | WCS-C Recorder |  |  |
| 55.21 | -129.14 | 21/11/2015 | 25/03/2016 | 125 | | | WCS-C Recorder |  |  |
| 59.43 | -126.10 | 25/09/2015 | 24/04/2016 | 212 | | | WCS-C Recorder |  |  |
| 50.52 | -121.72 | 26/11/2014 | 17/04/2015 | 142 | | | WCS-C Recorder |  |  |
| 50.83 | -121.88 | 4/11/2014 | 18/04/2015 | 165 | | | WCS-C Recorder |  |  |
| 50.63 | -121.86 | 9/12/2014 | 2/3/2015 | 83 | | | WCS-C Recorder |  |  |
| 50.28 | -115.85 | 31/10/2013 | 25/03/2014 | 145 | | | WCS-C Recorder |  |  |
| 50.28 | -115.85 | 21/09/2012 | 26/03/2013 | 186 | | | WCS-C Recorder |  |  |
| 55.06 | -129.47 | 14/10/2014 | 15/03/2015 | 152 | | | WCS-C Recorder |  |  |
| 49.30 | -116.76 | 17/11/2012 | 14/03/2013 | 117 | | | WCS-C Recorder |  |  |
| 49.01 | -118.35 | 17/12/2011 | 14/03/2012 | 88 | | | WCS-C Recorder |  |  |
| 49.78 | -119.74 | 23/10/2013 | 19/04/2014 | 178 | | | WCS-C Recorder |  |  |
| 49.08 | -116.58 | 17/11/2012 | 27/04/2013 | 161 | | | WCS-C Recorder |  |  |
| 49.45 | -119.56 | 15/06/2012 | 31/08/2012 | 77.5 | | | WCS-C Recorder |  |  |
| 49.44 | -119.57 | 16/11/2012 | 23/03/2013 | 127 | | | WCS-C Recorder |  |  |
| 49.91 | -126.65 | 24/10/2014 | 1/3/2015 | 128 | | | WCS-C Recorder |  |  |
| 57.90 | -131.17 | 4/10/2015 | 20/03/2016 | 168 | | | WCS-C Recorder |  |  |
| 49.76 | -124.57 | 3/12/2015 | 4/3/2016 | 92 | | | WCS-C Recorder |  |  |
| 59.57 | -133.70 | 6/10/2015 | 2/5/2016 | 209 | | | WCS-C Recorder |  |  |
| 54.07 | -131.80 | 30/12/2014 | 4/3/2015 | 64 | | | WCS-C Recorder |  |  |
| 53.58 | -124.79 | 18/10/2014 | 11/3/2015 | 144 | | | WCS-C Recorder |  |  |
| 49.30 | -119.55 | 30/12/2013 | 4/3/2014 | 64 | | | WCS-C Recorder |  |  |

**Table S2. Spatial body mass data for *Myotis lucifugus.*** Locations were obscured to the centre of the raster cell from which the data was taken.

| Latitude | Longitude | Mass (g) | Reference |
| --- | --- | --- | --- |
| 39.40 | -105.47 | 8.5 | VertNet |
| 39.51 | -121.55 | 7 | VertNet |
| 39.55 | -107.79 | 5.9 | VertNet |
| 39.72 | -96.64 | 7.3 | VertNet |
| 40.41 | -121.37 | 7 | VertNet |
| 40.63 | -105.15 | 7.7 | VertNet |
| 42.66 | -77.96 | 8.8 | VertNet |
| 42.69 | -77.96 | 6.9 | VertNet |
| 43.65 | -108.21 | 5.1 | VertNet |
| 43.99 | -75.93 | 14.5 | VertNet |
| 44.00 | -75.99 | 7.35 | VertNet |
| 44.03 | -76.05 | 9 | VertNet |
| 44.38 | -108.04 | 6.45 | VertNet |
| 44.54 | -89.56 | 8.2 | VertNet |
| 44.80 | -106.95 | 6.9 | VertNet |
| 44.88 | -107.26 | 5.4 | VertNet |
| 45.30 | -93.58 | 9.23 | VertNet |
| 47.91 | -122.10 | 6.45 | VertNet |
| 48.76 | -122.49 | 6.73 | VertNet |
| 57.76 | -152.52 | 6 | VertNet |
| 58.33 | -134.60 | 6.53 | VertNet |
| 58.33 | -134.60 | 6.65 | VertNet |
| 58.69 | -156.66 | 6.75 | VertNet |
| 59.45 | -135.32 | 7.08 | VertNet |
| 59.45 | -135.32 | 7.43 | VertNet |
| Latitude | Longitude | Mass (g) | Reference |
| 59.45 | -135.33 | 7.5 | VertNet |
| 59.50 | -135.26 | 8.04 | VertNet |
| 64.84 | -147.72 | 5.6 | VertNet |
| 64.85 | -148.05 | 5.4 | VertNet |
| 59.52 | -112.22 | 8.97 | WCS Canada |
| 60.05 | -112.68 | 9.91 | WCS Canada |
| 59.74 | -112.22 | 9.63 | WCS Canada |
| 59.75 | -112.20 | 9.94 | WCS Canada |
| 47.12 | -111.00 | 8.22 | Haase et al. 2019 |
| 41.81 | -111.62 | 6.72 | Haase et al. 2019 |
| 53.03 | -117.33 | 11.21 | Schowalter 1980 |
| 45.30 | -76.90 | 11.1 | McGuire et al. 2009 |
| 47.40 | -80.44 | 9.55 | Fenton 1970 |
| 53.10 | -99.16 | 12.56 | McGuire et al. 2016 |
| 43.24 | -73.04 | 9.06 | Kunz and T. H. Kunz 1987 |
| 43.24 | -73.04 | 9.19 | Kunz et al. 1998 |
| 45.30 | -76.90 | 10 | Fenton 1970 |
| 48.98 | -87.40 | 10.3 | Fenton 1970 |
| 46.29 | -81.86 | 9.5 | Fenton 1970 |
| 44.93 | -110.67 | 7.76 | Johnson et al. 2016 |
| 60.26 | -133.34 | 11.34 | WCS Canada Yukon |
| 60.36 | -134.59 | 8.73 | WCS Canada Yukon |

**Table 3 Summary of published literature reporting ambient temperatures observed in *Myotis lucifugus* hibernacula.** Sources with a single estimate typically represent the reported mean observed temperature; multiple estimates represent observations at multiple sites.

| Reference | State or  Province | Number of estimates |
| --- | --- | --- |
| Hitchcock 1949 | ON, QC | 3 |
| Layne 1958 | IL | 1 |
| Pearson 1962 | IL | 1 |
| Davis and Hitchcock 1965 | NY | 1 |
| Henshaw and Folk 1966 | KY | 1 |
| Martin et al. 1966 | NY | 1 |
| Fenton 1970 | ON | 1 |
| Fenton 1972 | ON | 1 |
| McManus 1974 | NJ | 1 |
| Brack 2007 | OH | 1 |
| Boyles et al. 2007 | OH | 2 |
| Langwig et al. 2012 | NY | 10 |
| Vanderwolf et al. 2012 | NB | 1 |
| Storm and Boyles 2011 | NY | 1 |
| Jonasson and Willis 2012 | MB | 4 |
| Kurta and Smith 2014 | MI | 1 |
| Reimer et al. 2014 | AB | 1 |

**Table S4. Model selection by AIC for alternative spatial models.** Terms included degrees of latitude North (Northing), elevation as represented by a digital elevation model (DEM), number of days in frost per year ($\text{Day}\text{s}_{\text{frost}}$), number of days below freezing during a year ($\text{Day}\text{s}_{\text{freezing}}$), the number of days outside of the growing season ($\text{Day}\text{s}_{\text{ngrow}}$), and the original a priori estimate from Hayman et al. 2016 (Original). Best model values are italicized and $\text{ΔAICc}$ is the difference between the respective model and the corresponding top model.

|  | Hibernation Duration | | Body Mass | |
| --- | --- | --- | --- | --- |
| Covariate Structure | AICc | ΔAIC | AICc | ΔAIC |
| $\text{Original}$ | 508.35 | 19.06 | 167.46 | 4.89 |
| $\text{DEM}$ | 523.01 | 33.72 | 172.73 | 10.16 |
| $\text{Day}\text{s}_{\text{freezing}}$ | 494.90 | 5.61 | 163.59 | 1.03 |
| $\text{Day}\text{s}_{\text{frost}}$ | 490.02 | 0.73 | 164.22 | 1.66 |
| $\text{Day}\text{s}_{\text{ngrow}}$ | 507.77 | 18.48 | 168.58 | 6.02 |
| $\text{Northing}$ | 515.44 | 26.16 | 172.24 | 9.67 |
| $\text{Northing}\text{ }\text{+ Original}$ | 510.36 | 21.07 | 167.97 | 5.40 |
| $\text{Northing}\text{ }\text{+ DEM}$ | 514.68 | 25.39 | 174.67 | 12.11 |
| $\text{Northing}\text{ }\text{+}\text{ }\text{Day}\text{s}_{\text{freezing}}$ | 497.05 | 7.76 | *162.57* | *0.00* |
| $\text{Northing}\text{ }\text{+ Day}\text{s}_{\text{frost}}$ | 491.46 | 2.18 | 166.65 | 4.08 |
| $\text{Northing}\text{ }\text{+}\text{ }\text{Day}\text{s}_{\text{ngrow}}$ | 509.83 | 20.55 | 169.85 | 7.28 |
| $\text{Northing}\text{ }\text{+ DEM}\text{ }\text{+ Original}$ | 512.59 | 23.30 | 167.63 | 5.07 |
| $\text{Northing}\text{ }\text{+ DEM}\text{ }\text{+ Day}\text{s}_{\text{freezing}}$ | 499.17 | 9.88 | 164.32 | 1.76 |
| $\text{Northing}\text{ }\text{+ DEM}\text{ }\text{+}\text{ }\text{Day}\text{s}_{\text{frost}}$ | *489.29* | *0.00* | 164.78 | 2.21 |
| $\text{Northing}\text{ }\text{+ DEM}\text{ }\text{+}\text{ }\text{Day}\text{s}_{\text{ngrow}}$ | 511.95 | 22.67 | 168.05 | 5.48 |
|  |  |  |  |  |

**Table S5.** **Summary of spatial products created for *Myotis lucifugus.*** Hibernation duration was predicted in days and defined as the annual period during which bats must hibernate to survive. Pre-hibernation body mass and initial fat mass were predicted from GLM models. Fat required refers to the predicted amount of fat required to survive the duration of winter hibernation. Survival was defined as the predicted fat required to survive hibernation – the predicted body fat available for bats going into hibernation and/or the predicted maximal number of days that a bat could hibernate – the duration of winter. Negative values represent a predicted shortfall in the fat required to survive hibernation while positive values indicate a surplus of fat stores remaining at emergence from hibernation. Finally, the % Increased represents the increased fat required to survive hibernation between infected and uninfected bats calculated as the difference between predicted fat use of the infected and healthy individuals divided by the healthy multiplied by 100.

| Results Layer | Units | Environmental Conditions | Pd Infection | Median | Mean | SD | 5% | 95% | Minimum | Maximum |
| --- | --- | --- | --- | --- | --- | --- | --- | --- | --- | --- |
| Hibernation Duration | days | - | - | 178.95 | 169.17 | 45.35 | 79.70 | 224.40 | 0.00 | 288.34 |
| Body Mass | grams | - | - | 8.64 | 9.14 | 1.84 | 7.04 | 12.52 | 4.53 | 23.27 |
| Body Fat | grams | - | - | 2.32 | 2.61 | 1.10 | 1.36 | 4.63 | 0.00 | 11.05 |
| Fat Required | grams | 4x98 | No | 0.48 | 0.45 | 0.12 | 0.21 | 0.60 | 0.00 | 0.77 |
| Fat Required | grams | 4x98 | Yes | 1.22 | 1.16 | 0.45 | 0.35 | 1.77 | 0.00 | 2.71 |
| Fat Required | grams | Best A. x 98 | No | 0.49 | 0.49 | 0.21 | 0.28 | 0.60 | 0.00 | 7.28 |
| Fat Required | grams | Best A. x 98 | Yes | 1.21 | 1.20 | 0.40 | 0.51 | 1.77 | 0.00 | 7.28 |
| Survival Capacity | grams | 4x98 | No | 1.84 | 2.16 | 1.01 | 1.07 | 4.05 | -0.50 | 10.67 |
| Survival Capacity | grams | 4x98 | Yes | 1.22 | 1.45 | 0.76 | 0.61 | 2.93 | -1.36 | 10.21 |
| Survival Capacity | grams | Best A. x 98 | No | 1.82 | 2.12 | 1.02 | 1.00 | 4.05 | -3.17 | 10.47 |
| Survival Capacity | grams | Best A. x 98 | Yes | 1.11 | 1.41 | 0.93 | 0.35 | 3.33 | -3.26 | 9.90 |
| Survival Capacity | days | 4x98 | No | 181.05 | 190.83 | 45.35 | 135.60 | 280.30 | 71.66 | 370.79 |
| Survival Capacity | days | 4x98 | Yes | 45.63 | 55.41 | 45.35 | 0.19 | 144.88 | -63.76 | 235.37 |
| Survival Capacity | days | Best A. x 98 | No | 181.05 | 188.79 | 49.37 | 134.22 | 280.07 | -175.95 | 370.79 |
| Results Layer | Units | Environmental Conditions | Pd Infection | Median | Mean | SD | 5% | 95% | Minimum | Maximum |
| Survival Capacity | days | Best A. x 98 | Yes | 45.38 | 46.86 | 32.55 | 0.66 | 102.67 | -175.95 | 182.56 |
| Increased Expenditure | % | 4x98 | - | 154.42 | 145.18 | 41.57 | 62.66 | 195.26 | 0.00 | 251.46 |
| Increased Expenditure | % | Best A. x 98 | - | 147.11 | 142.42 | 36.52 | 72.09 | 192.08 | 0.00 | 269.57 |

*
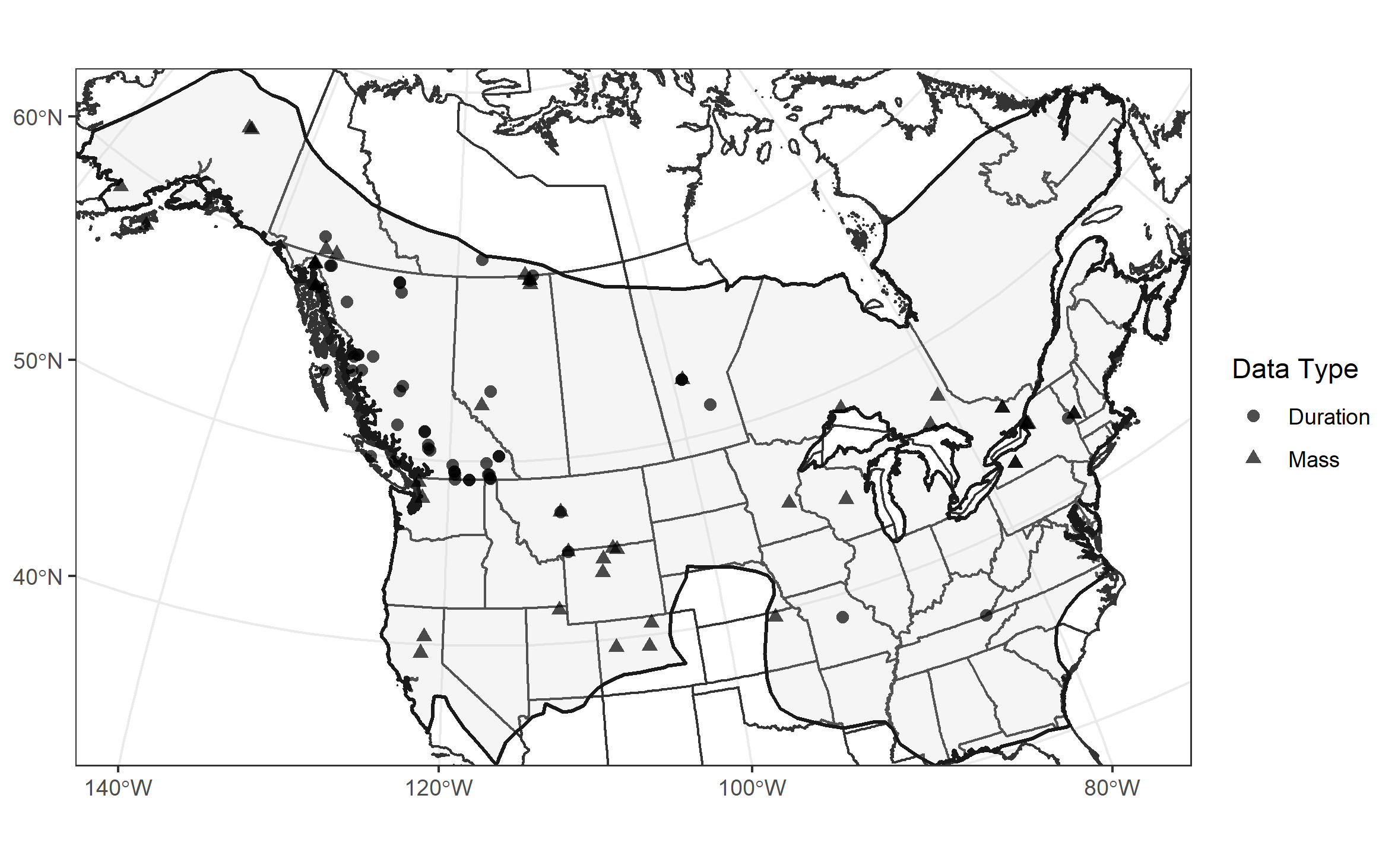
*

**Figure S1.** **Locations of *Myotis lucifugus* body mass and hibernation duration data across temperate North America.** Records of the duration of hibernation (o), pre-hibernation body mass (Δ) and the reported species range via IUCN (IUCN 2016); black outline with grey shaded interior)


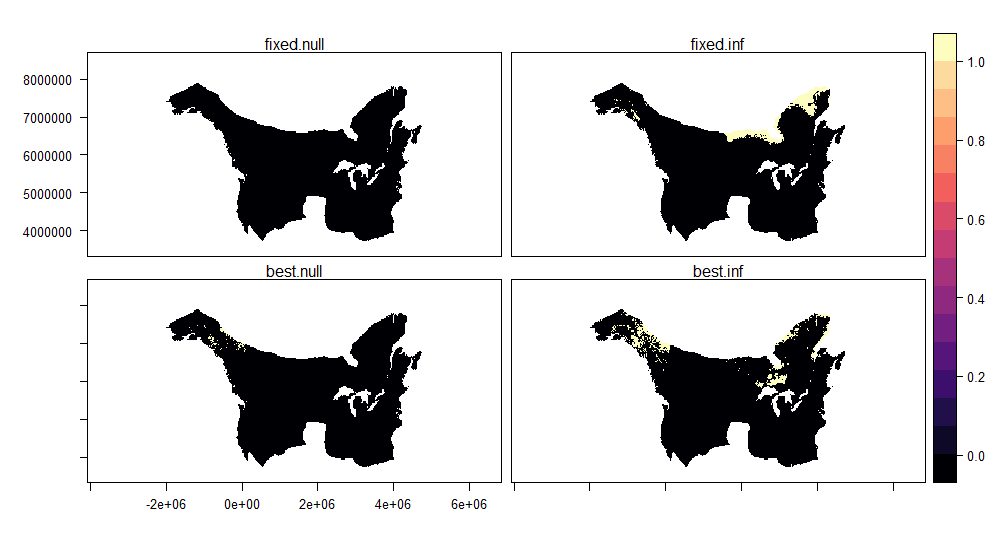


**Figure S2. Binary maps of predicted healthy (left column) and WNS-impacted (right column) *Myotis lucifugus* survival.** Yellow pixels indicate insufficient fat stores to survive the duration of winter while black pixels indicate survival. Top row: Fixed roosting conditions of 4°C and 98% relative humidity. Bottom row: best available microclimate conditions.


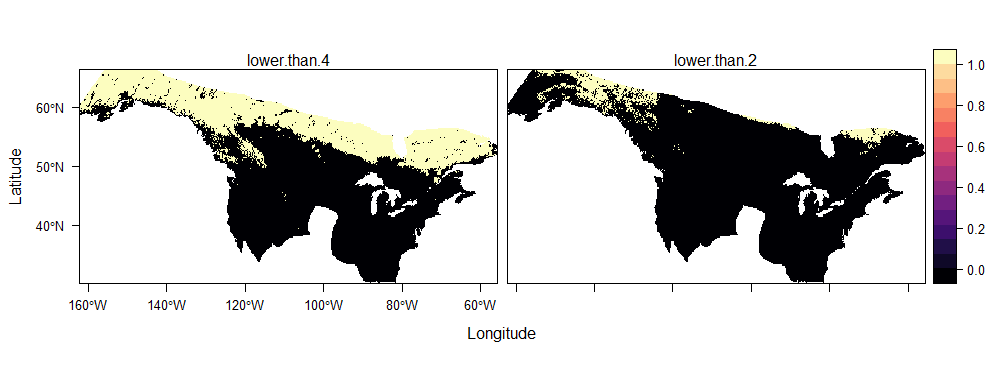


**Figure S3.** **Predicted best available roosting temperatures**. Left: yellow pixels indicate areas where the best available temperature is below 4°C. Right: yellow pixels indicate where temperatures were predicted to be below 2°C.


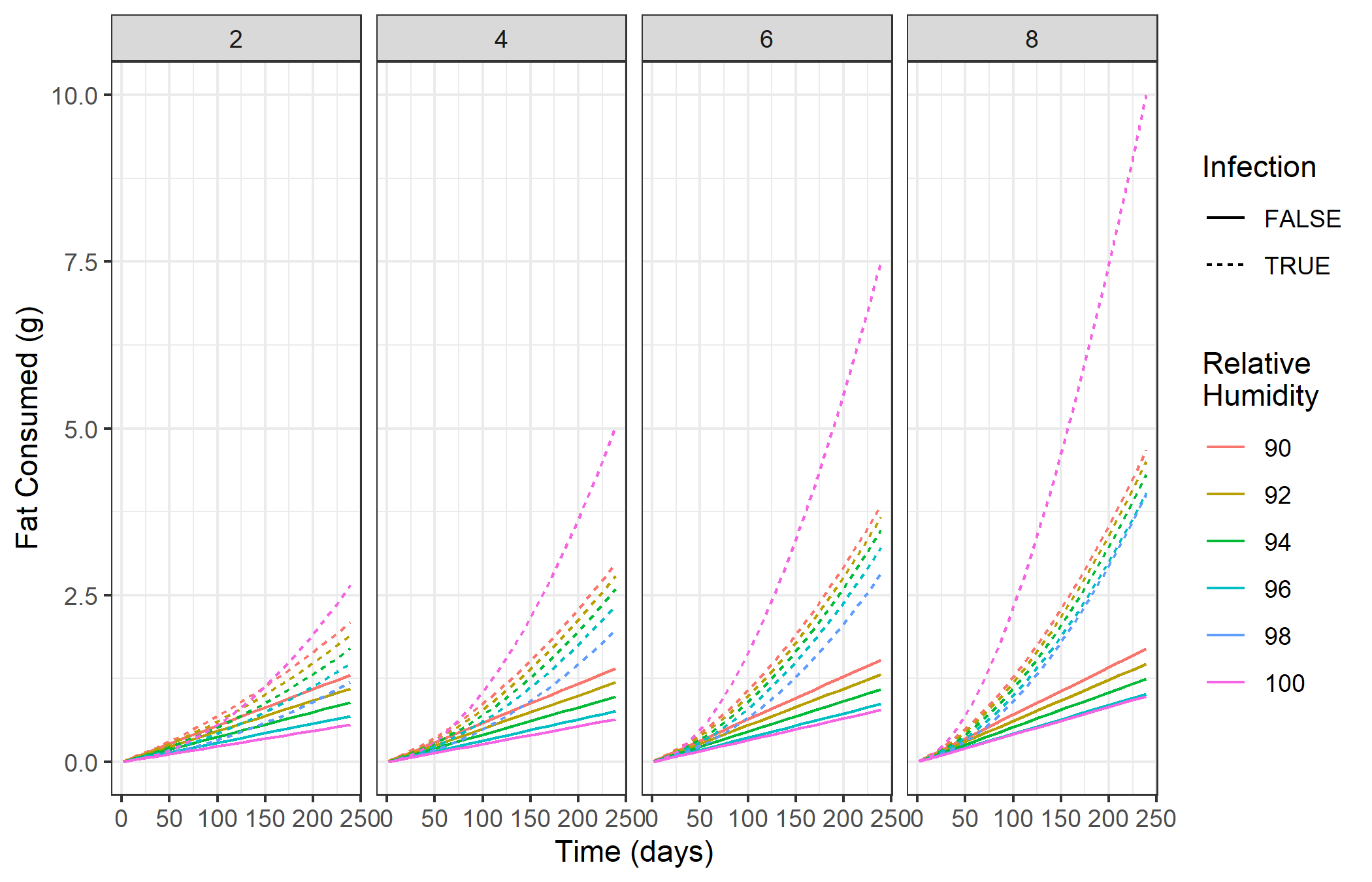


**Figure S4.** **Rate of energy expenditure at various roosting conditions for *Myotis lucifugus***. Each panel is a unique temperature (°C), and relative humidity (%) is depicted by line colour. Infectious status is denoted by line type with solid lines representing uninfected bats and dashed lines represent bats infected with P. destructans.

**REFERENCES**

Boyles, J. G. et al. 2007. Energy availability influences microclimate selection of hibernating bats. - Journal of Experimental Biology 210: 4345–4350.

Brack, V. 2007. Temperatures and Locations Used by Hibernating Bats, Including Myotis sodalis (Indiana Bat), in a Limestone Mine: Implications for Conservation and Management. - Environmental Management 40: 739–746.

Brack Jr., V. and Twente, J. W. 1985. The duration of the period of hibernation of three species of vespertilinoid bats. I. Field studies. - Canadian Journal of Zoology 63: 2952–2954.

Czenze, Z. J. and Willis, C. K. R. 2015. Warming up and shipping out: arousal and emergence timing in hibernating little brown bats (Myotis lucifugus). - Journal of Comparative Physiology B 185: 575–586.

Davis, W. H. and Hitchcock, H. 1965. Biology and migration of the bat,. - Journal of Mammalogy 46: 296–313.

Fenton, M. 1970. Population studies of *Myotis lucifugus* (Chiroptera: Vespertilionidae) in Ontario. - Life Sciences Contributions Royal Ontario Museum 77: 1–34.

Fenton, M. B. 1972. Distribution and overwintering of Myotis leibii and Eptesicus fuscus (Chiroptera, Vespertilionidae) in Ontario. - Royal Ontario Museum.

Hayman, D. T. S. et al. 2017. Long-term video surveillance and automated analyses reveal arousal patterns in groups of hibernating bats. - Methods in Ecology and Evolution 8: 1813–1821.

Henshaw, R. E. and Folk, E. F. 1966. Relation of Thermoregulation to Seasonally Changing Microclimate in Two Species of Bats (Myotis lucifugus and M. sodalis). - Physiological Zoology 39: 223–236.

Hitchcock, H. b. 1949. Hibernation of bats in southeastern Ontario and adjacent Quebec. - Canadian Field Naturalist 63: 47–59.

IUCN 2016. Terrestrial Mammals Red List Spatial Data. - The IUCN Red List of Threatened Species in press.

Johnson, J. S. et al. 2016. Migratory and winter activity of bats in Yellowstone National Park. - Journal of Mammalogy 98: gyw175.

Jonasson, K. A. and Willis, C. K. R. 2012. Hibernation energetics of free-ranging little brown bats. - The Journal of Experimental Biology 215: 2141–2149.

Kunz, K. a. and T. H. Kunz 1987. Size of bats at birth and maternal investment during pregnancy. - Symposia of the Zoological Society of London 57: 257–261.

Kunz, T. H. et al. 1998. Changes in body mass and fat reserves in pre-hibernating little brown bats (Myotis lucifugus). - Ecoscience 5: 8–17.

Kurta, A. and Smith, S. M. 2014. Hibernating Bats and Abandoned Mines in the Upper Peninsula of Michigan. - Northeastern Naturalist 21: 587–605.

Langwig, K. E. et al. 2012. Sociality, density-dependence and microclimates determine the persistence of populations suffering from a novel fungal disease, white-nose syndrome. - Ecology Letters 15: 1050–1057.

Layne, J. N. 1958. Notes on Mammals of Southern Illinois. - American Midland Naturalist 60: 219.

Martin, R. L. et al. 1966. Observations on Hibernation of Myotis Subulatus. - Journal of Mammalogy 47: 348–349.

McGuire, L. P. et al. 2009. Effect of age on energy storage during prehibernation swarming in little brown bats (Myotis lucifugus). - Canadian Journal of Zoology 87: 515–519.

McGuire, L. P. et al. 2016. No evidence of hyperphagia during prehibernation in a northern population of little brown bats ( *Myotis lucifugus* ). - Canadian Journal of Zoology 94: 821–827.

McManus, J. J. 1974. Activity and Thermal Preference of the Little Brown Bat, Myotis lucifugus, during Hibernation. - Journal of Mammalogy 55: 844–846.

Norquay, K. J. O. and Willis, C. K. R. 2014. Hibernation phenology of Myotis lucifugus. - Journal of Zoology 294: 85–92.

Pearson, E. W. 1962. Bats Hibernating in Silica Mines in Southern Illinois. - Journal of Mammalogy 43: 27.

Reimer, J. P. et al. 2014. Bat Activity and Use of Hibernacula in Wood Buffalo National Park, Alberta. - Northwestern Naturalist 95: 277–288.

Schowalter, D. B. 1980. Swarming, Reporduction and Early Hibernation of Myotis lucifugus and M. volans in Alberta, Canada. - Journal of Mammalogy 61: 350–354.

Storm, J. J. and Boyles, J. G. 2011. Body temperature and body mass of hibernating little brown bats Myotis lucifugus in hibernacula affected by white-nose syndrome. - Acta Theriologica 56: 123–127.

Vanderwolf, K. J. et al. 2012. Bat populations and cave microclimate prior to and at the outbreak of white-nose syndrome in New Brunswick. - Canadian Field-Naturalist 126: 125–134.
